# Taming the beasts inside

**DOI:** 10.7554/eLife.67634

**Published:** 2021-03-23

**Authors:** Erica P Ryu, Emily R Davenport

**Affiliations:** 1 Department of Biology, Pennsylvania State UniversityUniversity ParkUnited States; 2 Institute for Computational and Data Sciences, Pennsylvania State UniversityUniversity ParkUnited States; 3 Huck Institutes of the Life Sciences, Pennsylvania State UniversityUniversity ParkUnited States

**Keywords:** gut microbiota, domestication, canid, mouse, human, industrialization, Human, Mouse, Rat, Other

## Abstract

Changes in diet associated with domestication may have shaped the composition of microbes found in the guts of animals.

**Related research article** Reese AT, Chadaideh KS, Diggins CE, Schell LD, Beckel M, Callahan P, Ryan R, Thompson ME, Carmody RN. 2021. Effects of domestication on the gut microbiota parallel those of human industrialization. *eLife* **10**:e60197. doi: 10.7554/eLife.60197

What do cats, dogs and cows have in common? They have all been domesticated. In other words, humans have selectively bred their wild ancestors for traits suited to our needs, whether for companionship, agriculture or science.

Domestication leads to both genetic and ecological changes. Desirable, genetically encoded traits, such as tameness or greater muscle mass, are selected for over generations of breeding. The ecological changes associated with domestication include increased population density, different habitats and changes to diet. While the impact of these factors on various animal traits has been studied extensively, relatively little is known about their effect on the microbiome (that is, the microbes that live inside and on these animals). Does domestication influence the microbiome? If so, are genetic or ecological factors driving these changes?

Now, in eLife, Aspen Reese, Rachel Carmody and colleagues at Harvard University, the Wildlife Science Center in Minnesota, and the University of New Mexico report that domestication does indeed influence the microbiome (Reese et al., 2021). The researchers compared the gut microbiomes across pairs of domesticated animals and their wild counterparts, including cattle and bison, dogs and wolves, and laboratory and wild-caught mice. They found consistent shifts in the composition of the gut microbiomes within the pairs, suggesting that domestication has an influence on the microbiome.

But what underlying factors are driving this shift? Is it due to the genetic changes that resulted from domestication, the ecological changes, or both? To address this, Reese et al. performed clever diet-swap experiments in two systems: laboratory vs. wild mice, and dog vs. wolf. Specifically, wild animals were fed the diets of their domesticated counterparts, and vice versa. The researchers found that this change in diet led to significant changes in the gut microbiome. For example, the microbiomes of wolves fed commercial dog food shifted towards a dog-like state. Dogs fed raw carcasses – aside from having the best week ever – saw their microbiomes shift even more dramatically towards a wolf-like state (Figure 1). These experiments demonstrate that ecological shifts – specifically changes in diet – can have a major impact on the microbiome.

**Figure 1.**
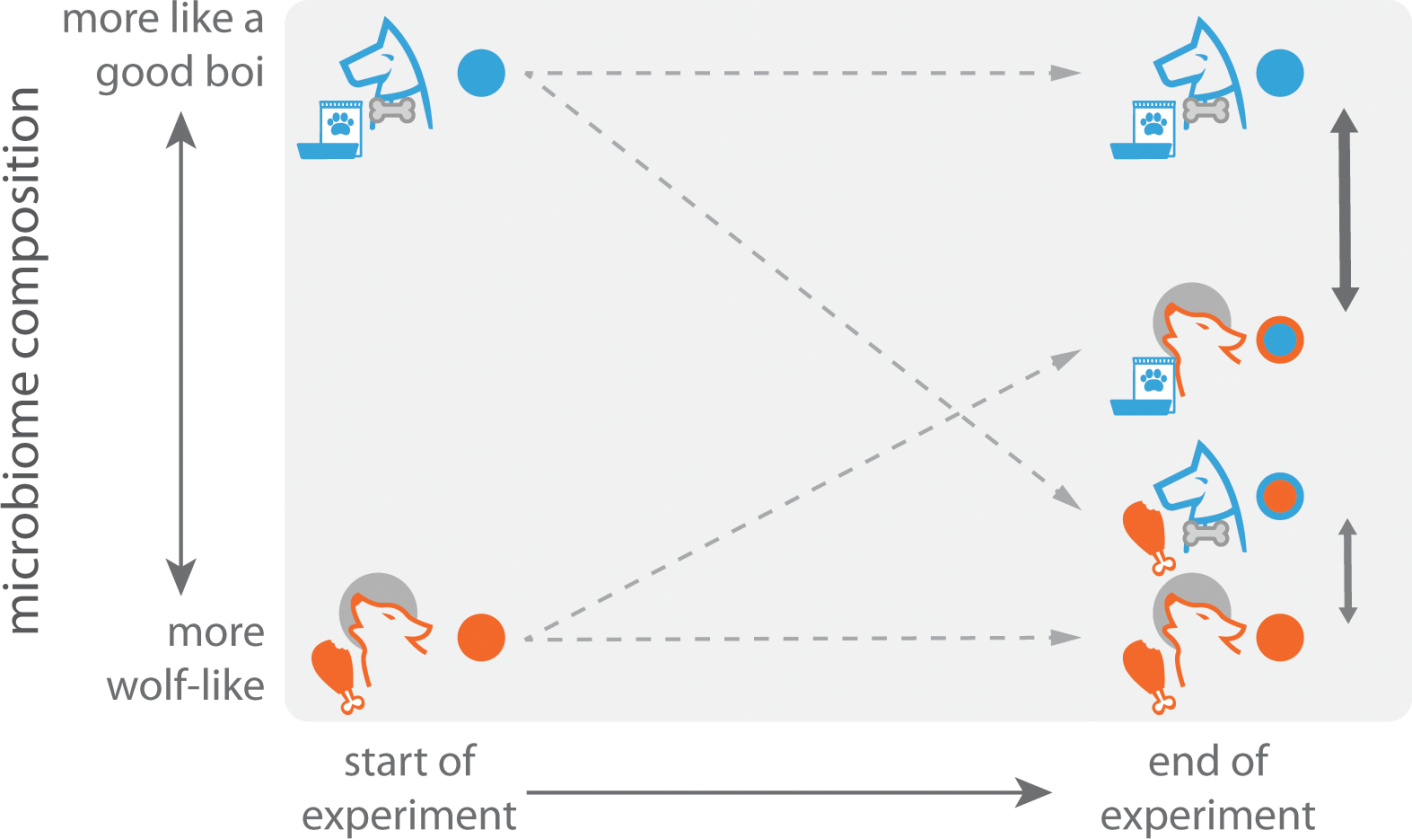
A diet-swap experiment with dogs and wolves. During a seven-day experiment, the natural diets of dogs (commercial dog chow; blue) and wolves (carcasses; orange) were swapped, and their gut microbiomes measured at the end of the experiment. The gut microbiomes of control samples of dogs and wolves that were fed their natural diets were also measured. At the end of the experiment, the microbiomes of wolves eating dog chow more closely resembled the microbiomes of dogs than the microbiomes of wolves eating their natural diet. An even stronger effect was observed for dogs. This could be due to the microbiomes of dogs being more plastic as a result of consuming a variable omnivorous diet, whereas wolves have a narrower carnivorous diet.

These results, and many others not covered here, open a slew of questions to explore. For example, while there are clear compositional shifts in the microbiome with domestication, are there individual microbes that are particularly important to this process? Would the purpose of domestication – such as agriculture, companionship or laboratory model – change which microbes were important? In addition to taxonomic composition, is there a signature of domestication in the microbial functions encoded by the microbiome?

While Reese et al. demonstrated the potential role of ecological factors in shaping the microbiome during domestication, it remains unclear whether genetic factors also play a role. Domestication impacts numerous, genetically encoded morphological and behavioral traits. Interestingly, the genes selected for during domestication often influence multiple, seemingly unrelated, traits through a process called pleiotropy. For example, when selecting for tameness in domesticated foxes, other unexpected physical changes such as coat depigmentation and floppy ears were observed (Trut, 1999). This is due to deficits of neural crest cells (embryonic cells that serve as precursors for tissues all around the body [Wilkins et al., 2014]). Could these genetic variants also affect the gut microbiome, either directly or indirectly? This is certainly a possibility. A portion of the gut microbiome is determined by host genetics (Goodrich et al., 2014). Given that genetic variants associated with the microbiome can have pleiotropic effects, genes undergoing selection through domestication may also impact the microbiome (Benson et al., 2010).

One final open question is whether the changes in the composition of the microbiome that accompany domestication also have an impact on physiology. For example, domesticated dogs are more capable of digesting starch than their wild counterparts. This is due to an increased number of variants of the gene for amylase, an enzyme that is responsible for breaking down starches (Freedman et al., 2014). Amylase copy number is also associated with microbiome composition and functional capacity in humans. Transfer of the microbiome from an individual with high amylase numbers to a gnotobiotic mouse (a germ-free mouse) results in high body-fat composition, demonstrating the direct physiological impact the microbiome can have on the host (Poole et al., 2019). Knowing that microbes contribute to metabolism and nutrient absorption across numerous animals, it is possible that domestication-related shifts of specific microbes may alter the physiology of the host (Ramezani et al., 2018; Warnecke et al., 2007).

The work of Reese et al. is an impressive and comprehensive examination of the impact of domestication on the microbiome. It has the potential to spur numerous lines of research in specific microbes and genes involved in this association, and how microbial communities shift in response to environmental factors. So, the next time you go to pet your tamed wolf (aka: your dog), give some love to their tamed microbes as well.
